# FEM Simulations of Fatigue Crack Initiation in the Oligocrystalline Microstructure of Stents

**DOI:** 10.3390/ma16176003

**Published:** 2023-08-31

**Authors:** Galina Lasko, Siegfried Schmauder, Yitong Yang, Sabine Weiss, Kiarash Dogahe

**Affiliations:** 1 Institute for Materials Testing, Materials Science and Strength of Materials (IMWF), University of Stuttgart, Pfaffenwaldring 32, D-70569 Stuttgart, Germany; 2 Department of Physical Metallurgy and Materials Technology (MWT), Brandenburg University of Technology Cottbus-Senftenberg, Konrad-Wachsmann-Allee 17, D-03046 Cottbus, Germany

**Keywords:** stents, oligocrystals, cyclic tensile loading, cyclic bending loading, Wöhler curves for crack initiation, finite element analysis (FEA)

## Abstract

For over two decades, vascular stents have been widely used to treat clogged vessels, serving as a scaffold to enlarge the narrowed lumen and recover the arterial flow area. High-purity oligocrystalline austenitic steel is usually applied for the production of stents. Despite the popularity and benefit of stenting, it still may cause serious clinical adverse issues, such as in-stent restenosis and stent fracture. Therefore, the study of the mechanical properties of stents and in particular the prediction of their life cycles are in the focus of materials research. In our contribution, within the finite element method, a two-scale model of crack initiation in the microstructure of stents is elaborated. The approach is developed on the basis of the physically based Tanaka–Mura model (TMM), considering the evolution of shear bands during the crack initiation phase. The model allows for the analysis of the microstructure with respect to the life cycles of real materials. The effects of different loading conditions, grain orientation, and thickness of the specimen on Wöhler curves were analysed. It was found that the microstructural features of oligocrystals are very sensitive to different loading conditions with respect to their fatigue behaviour and play a major role in fatigue crack initiation. Different grain-orientation distributions result in qualitative and quantitative differences in stress distribution and in the number of cycles for crack initiation. It was found that presence of a neutral zone in the cut-out of the microstructure under three-point-bending loading conditions changes the qualitative and quantitative patterns of stress distribution and affects the number of cycles for crack initiation. It was found that under both tensile and bending loading conditions, thicker specimens require more cycles for crack initiation. The Wöhler curves for crack initiation in oligocrystalline microstructures of stents could be compared with the ones in the experiment, taking into account that for high cyclic fatigue (HCF), typically, more than 70% of the cycles refer to crack initiation. The developed numerical tools could be used for the material design of stents.

## 1. Introduction

According to the data of the World Health Organization, cardiovascular disease is the most common reason of death among people. For example, in 2019, this number reached over 18.56 million people, surpassing even the number of deaths caused by cancer and diabetes.

The cause of this disease is the deposition of cholesterol plaques on the walls of the arteries, which results in the narrowing of the lumen, which impedes the passage of blood flow and can lead to heart attack and death.

**Figure 1:**
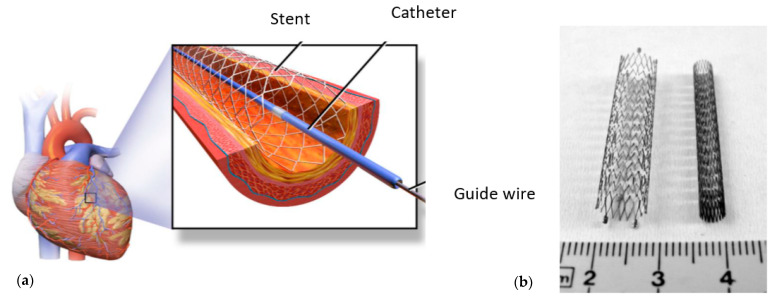
(**a**) Schematic representation of inserted stent in coronary artery; (**b**) coronary stents made from steel (image: Representational/Courtesy—Wikipedia).

One of the methods to solve the problem is to install stents in the place of the narrowing of the artery (Figure 1). Stents are medical devices that are used in narrowed or weakened arteries for providing support and improving blood flow. Once inserted, the lumen is expanded by the stent, leading to improved blood flow. Often, the method of balloon expansion is used to install the stent.

A stent is a woven, knitted, or braided cylindrical mesh structure made from stainless steel, nitrol, or chromic-cobalt alloy that is inserted in a diseased or contracted artery or vein to restore free blood flow by keeping its vessel open.

Certain alloys can be used to produce coronary stents. Stainless steel is the most common material used for stents. Its advantages include good X-ray visibility, fairly elastic and resistant consistency, and good biocompatibility. These qualities make this type of stent very suitable for balloon insertion and prevent sudden vessel closure.

Because stents are small objects and have complex geometries, it can be difficult to predict crack initiation using standard tests and specimens. One approach to predicting crack initiation in stents is computational modelling. Finite element analysis (FEA) can be used to simulate the mechanical behaviour of stents under different loading conditions and predict areas of stress concentration that will likely result in crack initiation.

FEA is an extremely useful tool that has proven to be effective and capable of providing a better and a more detailed understanding of fatigue and design. In [1], it has been demonstrated that both the percentage of artery expansion and the dimensions of the struts have an impact on fatigue behaviour after stent deployment.

There is a significant body of literature on the finite element analysis of the fatigue behaviour of stents [2,3]. The engineer can then use the analysis results to modify the design and prevent failure, without making and testing numerous physical devices. Most of the works dealing with stents are devoted to the simulation of the behaviour of different stent designs, biocompatibility, and tissue reactions [4,5,6]. In these works, the material is considered to be homogeneous without taking the microstructure of the strut into account. In others, like [5], the interplay of stent and balloon is analysed.

The results of computational fatigue approaches are available in most of the literature, for example, in [7,8]. Mostly, phenomenological models within the framework of J_2_-plasticity are formulated, but there are also damage accumulation models [9].

Most of the models concentrate on the propagation of existing cracks [10], and very little is known on the crack initiation problem numerically [11]. Nevertheless, in the microstructure of stents, crack initiation prediction plays a major role in the estimation of the life cycle of the material.

Atomistic simulations of fatigue crack initiation are promising for a very accurate representation of microscale mechanisms [12]. But the problem with these atomistic models is that they are quite computationally expensive and only cover a limited simulation domain. An alternative approach is the description of fatigue crack initiation and propagation using phase-field modelling [13]. In [14], crack initiation and propagation are simulated in combination with the application of the Smith–Watson–Topper damage parameter. However, phase-field models cannot describe the microstructural features as done, for example, within the framework of the crystal-plasticity (CP) approach [15].

Nevertheless, the computational costs of CP models for fatigue crack initiation are very high, especially for complex structures and under complex boundary conditions; therefore, they have been applied, up to now, to a limited number of cases (2D cases, tensile loading conditions, etc.).

Therefore, it is necessary to develop alternative models for the prediction of the fatigue behaviour of a material taking into account the microstructure. One of the alternative approaches is the one presented in our contribution, combining the physically based Tanaka–Mura model in the framework of the FEM software ABAQUS 2018.

In a great body of publications, much attention has been devoted to the simulations of crack propagation under fatigue. For example, in [16], a crack is simulated by introducing a zero-thickness cohesive contact element coupled with a damage parameter that was developed from material observations of strain-controlled fatigue experiments. Among the limitations of this algorithm in the current state is mesh dependency, as it affects the path of the crack’s growth. The cracks can only grow along the element edges.

In order to avoid such mesh-dependent difficulties, a novel approach known as extended finite element method (XFEM) has been developed to facilitate the modelling of crack growth problems by using the partition of unity enrichment of finite elements [17]. With the application of this method, crack advancement is defined in XFEM independently of the initial FE mesh. The crack is also simulated using the element elimination technique (EET), in which the element is simply deleted from the mesh upon achieving the damage criterion [18].

As an alternative to crack simulations with CZM, XFEM, and EET, in the present approach, the crack is introduced in the shell model by creating a seam along the predefined slip band in which the fracture criterion is achieved. The introduction of the crack is accompanied by the remeshing of the area of the grain.

The main purpose of this work is to present a methodology to estimate the fatigue crack initiation life of oligocrystals using the T-M model incorporated in the FEM framework and taking into account the microstructure as close as possible to the real one. In our work, we concentrate on the FEM simulations of the high-cyclic-fatigue behaviour of the oligocrystalline material of stents under tensile and bending loading conditions with explicit consideration of the microstructure.

This paper is organized as follows: In the following section, Section 2, the material and methods are presented. The physically based Tanaka–Mura (T-M) model and the computational model are also presented in this section. The results of simulations under tensile and bending loading conditions are contained in Section 3, considering the cases of tensile and bending loading conditions. At last, some concluding remarks close the paper in Section 4.

## 2. Materials and Methods

### 2.1. Material

The material used in this investigation was 316 LVM steel, from the Zapp Medical Alloys GmbH, Germany with yield stress 434 MPa and elastic modulus of 187.5 GPa. In Table 1, the chemical composition of 316 LVM steel, which includes some elements such as Cr, Mo, and Ni at low concentrations, is presented, while the physical properties of the frequently used stent material 316 LVM steel are presented in Table 2.

One more challenge in simulating fatigue crack initiation is the representation of the grain-level morphological details. Creating an accurate representation of the microstructure is crucial. Microstructures are complex and heterogeneous, consisting of various grain sizes. Capturing these features correctly is essential for accurate simulation.

In order to correctly simulate the initiation and growth of short cracks, it is necessary to consider the microstructure, which includes the morphology and orientation of grains at the micro-level.

**Figure 2:**
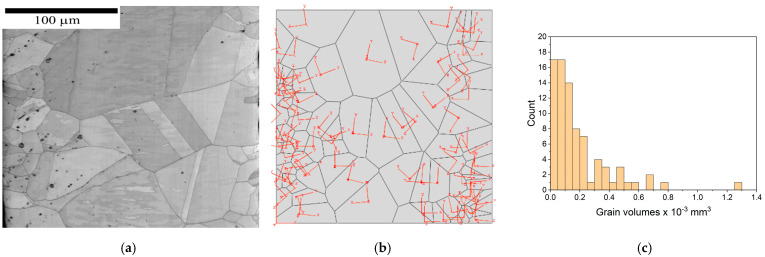
Cut-out of oligocrystalline microstructure (**a**) observed with SEM and (**b**) generated with Neper software; (**c**) grain-volume distribution from (**b**).

A 3D representative volume element of a microstructure containing 81 grains is represented in Figure 2a. For the generation of the microstructure, the software package Neper 4.6.0 [15] was used. From the experimental characterization of the grain structure using scanning electron microscopy images of the material (Figure 2a), the coordinates of the centres of the grains were determined, and with the application of tessellation software, a microstructure similar to the original one was generated (Figure 2b). In Figure 2c, the histogram of grain-volume distribution is presented. The volumes of each grain in the microstructure were obtained as output from ABAQUS.

The behaviour of the material investigated was considered to be elastic–plastic orthotropic behaviour. The input parameters for the model were critical resolved shear stress, fracture energy, Poisson’s ratio. The elastic constants used in the model were C_11_ = C_22_ = C_33_ = E(1 – ν)/(1 – ν – 2ν^2^) = 277.8 GPa; C_12_ = C_13_ = C_23_ = E ν/(1 – ν – 2ν^2^) = 136.8 GPa; und C_44_ = C_55_ = C_66_ = G = 70.3 GPa.

### 2.2. Mesh and Boundary Conditions

**Figure 3:**
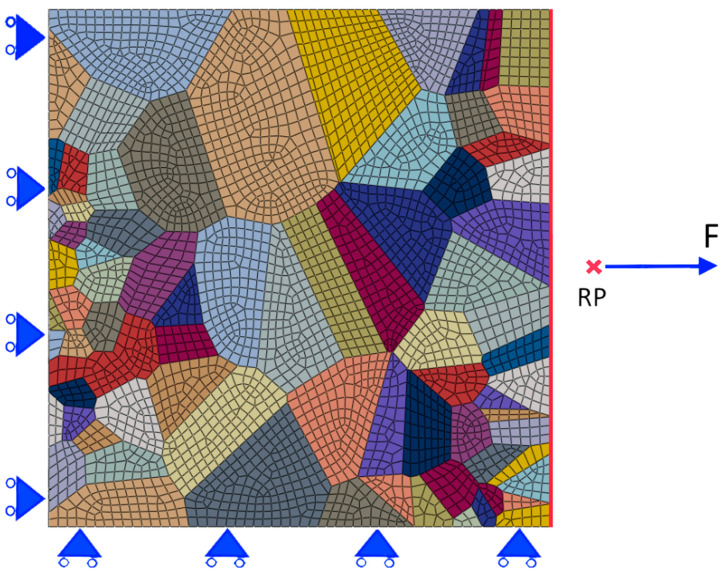
FE mesh and loading boundary conditions for the cut-out of the microstructure from Figure 2 under cyclic tensile loading (R = 0).

The cut-out of the microstructure was divided into a total of 3592 linear membrane elements (M3D4R and M3D3) (Figure 3). The mesh density was chosen based on a mesh convergence study. For the simulation of crack initiation, the physically based Tanaka–Mura (T-M) model was applied.

Simulations of fatigue crack initiation were performed on the cut-out (from Figure 2) of the microstructure under cyclic tensile loading conditions at stress ratio R = σ*_min_*/σ*_max_* = 0. The cut-out of the microstructure was divided into 3592 3D membrane elements. Shown in Figure 3 is the FE mesh of the cut-out of the microstructure and the applied tensile loading boundary conditions. X- and Y-symmetry boundary conditions were imposed on the left-hand side and bottom side of the microstructure, respectively.

The right-hand side of the specimen (red line) was constrained using an equation with reference point N, and concentrated force was applied to the reference point. For each grain, 3D orientation was assigned using the generation of three Euler angles with a generator of random numbers. For simplification, the projections of the 3D orientation are plotted in Figure 2b.

### 2.3. Tanaka—Mura Model

Modelling the processes of the early stages of fatigue crack nucleation and growth at the microstructure scale is an important emerging frontier, and this can support microstructure-sensitive estimations of minimum life cycles and can suggest a modification of the process route to alter the microstructure in ways that promote enhanced resistance to the formation of fatigue cracks [19].

In order to simulate the fatigue behaviour of the material, it is necessary to understand the mechanisms that facilitate crack initiation in a first step, and in a second one, the ones that facilitate crack propagation. Slip irreversibilities exist in a material and accumulate during fatigue loading. At the defect level, irreversibilities are a result of dislocations: annihilating, cross-slipping, penetrating precipitates, transmitting through grain boundaries, and piling up. These slip irreversibilities are the early signs of damage during cyclic loading, and dislocations subsequently form low-energy, stable structures as a means to accommodate the irreversible slip processes; dislocation density increases during cyclic forward and reverse loading, and the result is strain localization in a small region within the material, i.e., persistent slip bands and dislocation cells/bundles. Strain localization is a precursor to crack initiation.

This fundamental understanding is necessary to study persistent slip bands in Face-Centred Cubic (FCC) metals and alloys, including appropriate characterization, theory, and modelling. From this fundamental knowledge, both micromechanical and crystal-plasticity models, which are also reviewed, can be used to predict crack initiation.

**Figure 4:**
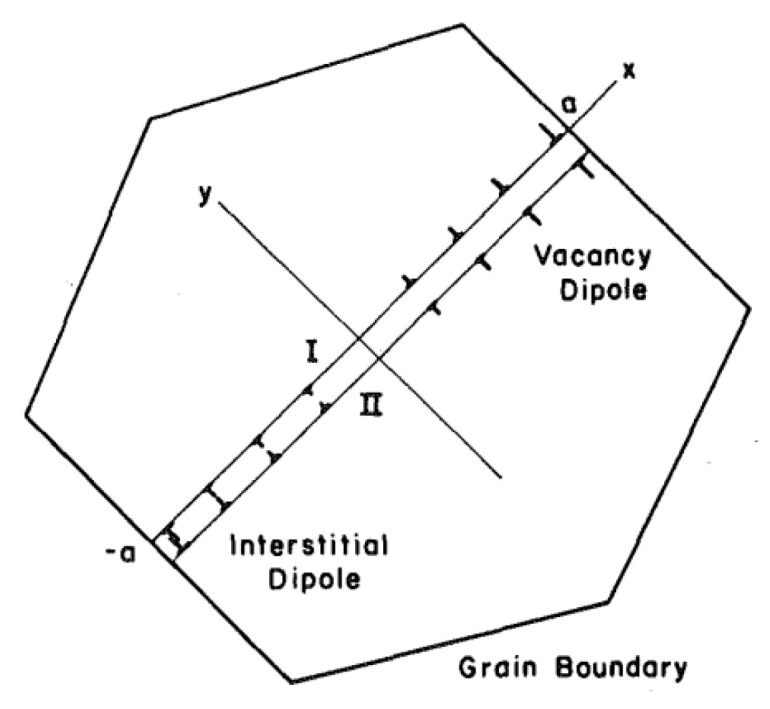
Dislocation motion in the most favourably oriented grain [10].

The study of fatigue crack initiation in metals was one of the most important technical topics in the 19^th^ century [11]. Although the mechanisms of crack initiation are still under discussion today, some useful models have been proposed to describe the microstructural evolution before crack initiation occurs. The Tanaka–Mura formulation is one of those models. It is a dislocation model at the microscale level and states that a micro-crack initiates along a slip band when the energy caused by dislocation accumulation reaches a threshold. This model considers fatigue crack initiation as the consequence of the agglomeration of dislocation dipoles, which creates the so-called persistent slip bands in each grain (Figure 4) [20,21]. Crack nucleation life *N_n_* can be determined as follows:

(1) $$N_{n}=\frac{8GW_{c}}{\pi (1-\nu ){d(∆\bar{\tau }-2CRSS)}^{2}}$$

where *G* is the shear modulus, *CRSS* is the critical resolved shear stress, *v* is Poisson’s ratio, *W_c_* is the specific fracture energy per unit area, *d* is the slip-band length size, and ∆$\bar{\tau }$ is the average shear-stress range on the slip-band length. The data for the material parameters used can be found in the literature [22].

The necessary parameters for the simulations of fatigue crack initiation are presented in Table 3.

Equation (1) presumes that micro-cracks form along the slip band of grains, depending on slip-band length d and the average shear-stress range on the slip band.

First published in 1981 by K. Tanaka and T. Mura, it has since become a frequently used tool for studying fatigue crack initiation in a wide range of materials, including ceramics, polymers, and metals.

In the paper by A. Brückner-Foit from 2006 [22], the model was applied for the prediction of crack initiation in martensitic steel. Crack initiation was modelled by placing the cracks at the centres of grains.

In the work by Jezernik [23,24], three improvements were added to the model: multiple slip bands where micro-cracks may appear were used in each grain; micro-crack coalescence was achieved by extending existing micro-cracks along grain boundaries; and segmented micro-crack generation was performed, whereby the crack is considered to propagate along segments of shear bands.

The number of cycles for crack initiation was derived from the formula for the energy necessary to create the crack.

### 2.4. Computational Model

The simulations were performed within the framework of the FE software suite ABAQUS CAE 2018. The tessellated microstructure, generated with Neper software [25], was input in the Sketch module of ABAQUS and meshed within the same software package. After applying boundary and loading conditions, the simulations of crack initiation for each stress amplitude were carried out using a plug-in in ABAQUS CAE software.

In each grain, the possible slip bands were determined. The critical resolved shear stress was assigned to the program. During fatigue loading, the slip-band segment, in which the shear stress achieved the critical value, was determined, and the crack was introduced in this segment by remeshing the whole mesh in this grain. The crack was created by introducing a seam in the mesh.

**Figure 5:**
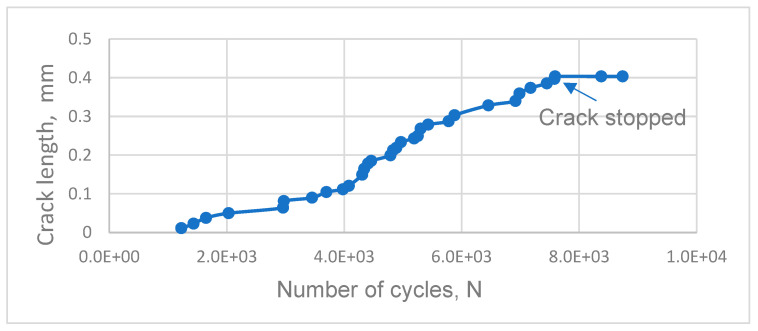
Dependence of crack length on the accumulated number of cycles for the case of stress amplitude σ_a_ = 900 MPa, the first distribution of grain orientation, and thickness d = 0.2 mm.

A detailed description of the simulations of fatigue crack initiation is contained in the works [26,27,28,29]. The number of cycles for crack initiation was determined using an iterative approach. At each iteration, the number of cycles for the initiation (arising) of a new segment of the crack was determined. At the same time, the total micro-crack length was recorded, and the velocity of crack propagation was calculated. After achieving the critical length of the nucleated micro-crack, when the crack stopped, as, for example, in Figure 5, the number of cycles was determined.

For the prediction of the Wöhler diagram for crack initiation, six cyclic stress amplitudes were considered in the numerical analyses: 429, 582, 656, 730, 891, and 990 MPa.

## 3. Results and Discussion

### 3.1. FEM Simulations of Crack Initiation in Oligocrystalline Microstructure under Cyclic Tensile Loading Conditions

It is known that a stent in a vessel is subjected to a complex loading condition. In order to check the model and to study the effect of different loading conditions, we consider the tensile loading conditions first.

**Figure 6:**
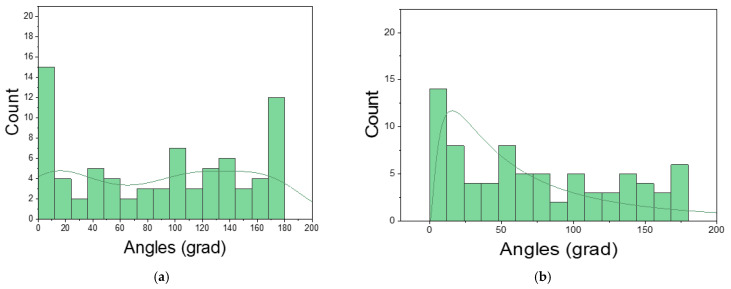
Two distributions of grain orientation: (**a**) the first distribution; (**b**) the second distribution.

Two distributions of grain orientation were analysed in our simulations (Figure 6a,b). The differences in grain orientation were considered the rotation of 2D coordinate systems around the z-axis (in the 2D case, the *z*-axis is perpendicular to the x-y plane).

The simulations were performed for different thickness values of the specimens, 0.06 mm and 0.2 mm, under tensile loading conditions. The thickness values and loads were chosen in such a manner to keep the applied stress in a comparable range in order to study how the different loading conditions affected the crack initiation life.

**Figure 7:**
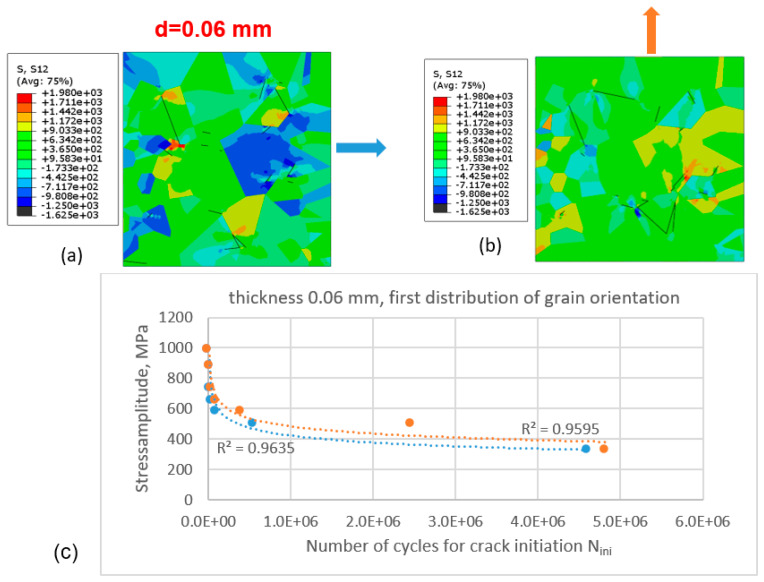
Initiation of fatigue cracks in the cut-out of the microstructure with the first distribution of grain orientation according to Figure 6a under cyclic tensile loading conditions for specimen thickness d = 0.06 mm: (**a**) shear-stress distribution and initiated cracks in the cut-out loaded in the x-direction and (**b**) in the y-direction; (**c**) Wöhler diagram for crack initiation for cases (**a**) (blue dashed line and filled circles—loading in the x-direction) and (**b**) (light-orange dashed line and filled circles—in the y-direction).

The shear-stress distributions under the applied stress amplitude σ_a_ = 1800 MPa after N cycles are presented in Figure 7 for two different directions of applied stress: the horizontal X-direction (Figure 7a) and the vertical Y-direction (Figure 7b). The elevated stress amplitude was selected for a more illustrative representation of the stress field, which shows a clearly pronounced grain structure.

The difference in stress distribution in the above two cases is clearly pronounced. The Wöhler curves for crack initiation are identical for higher stress values, and there is a difference in the number of cycles of one order of magnitude for lower values of stress amplitude. For the stress amplitude of 429 MPa and the loading of the cut-out of the microstructure in the vertical direction, the number of cycles is one order of magnitude higher than that for the same microstructural cut-out loaded in the perpendicular direction. It is clearly seen that the direction of loading can have a significant impact on the stress and strain distributions due to the anisotropic behaviour of the grains of oligocrystals. For example, under loading applied in the horizontal direction (or x-direction; Figure 7a), more negative shear-stress values are observed compared with the case in Figure 7b.

It is clearly seen that there is no difference in the number of cycles for stress amplitudes from 700 MPa to 1000 MPa in both cases. But for lower stress values, from 600 MPa to 323 MPa, the difference in the number of cycles is clearly pronounced.

**Figure 8:**
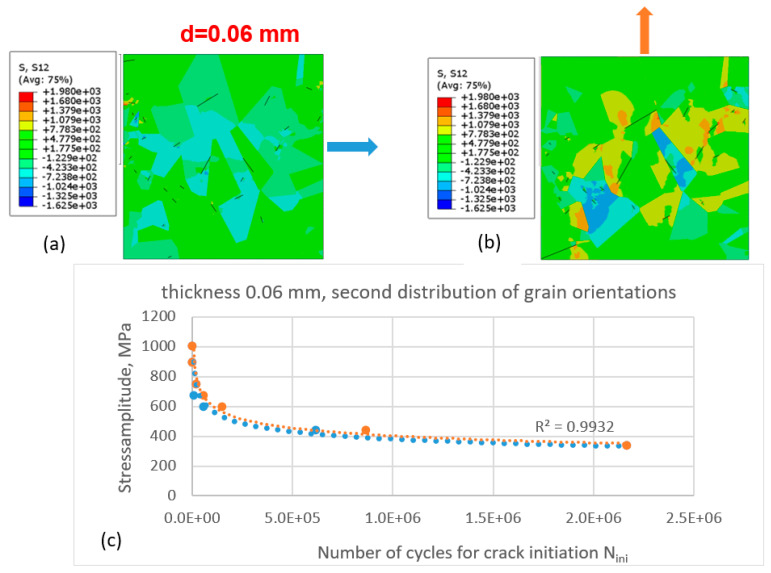
Initiation of fatigue cracks in the cut-out of the microstructure with the second distribution of grain orientation according to Figure 6b under cyclic tensile loading conditions for specimen thickness d = 0.06 mm: (**a**) shear-stress distribution and initiated cracks in the cut-out loaded in the x-direction and (**b**) in the y-direction; (**c**) Wöhler diagram for crack initiation for cases (**a**) (blue dashed line and filled circles—loading in the x-direction) and (**b**) (light-orange dashed line and filled circles—in the y-direction).

The simulations were performed for the same thickness of the specimen (Figure 8) and for the second grain-orientation distribution according to Figure 6b.

Compared with the case of the first grain-orientation distribution (Figure 7), for the second grain-orientation distribution, the difference in the number of cycles at the second segment of the Wöhler curve is smaller, while the best-fit lines for both sets of data points (matching the different directions of force application) are almost identical. One can see different qualitative and quantitative patters of shear-stress distribution compared with the first case (different distributions of grain orientation).

**Figure 9:**
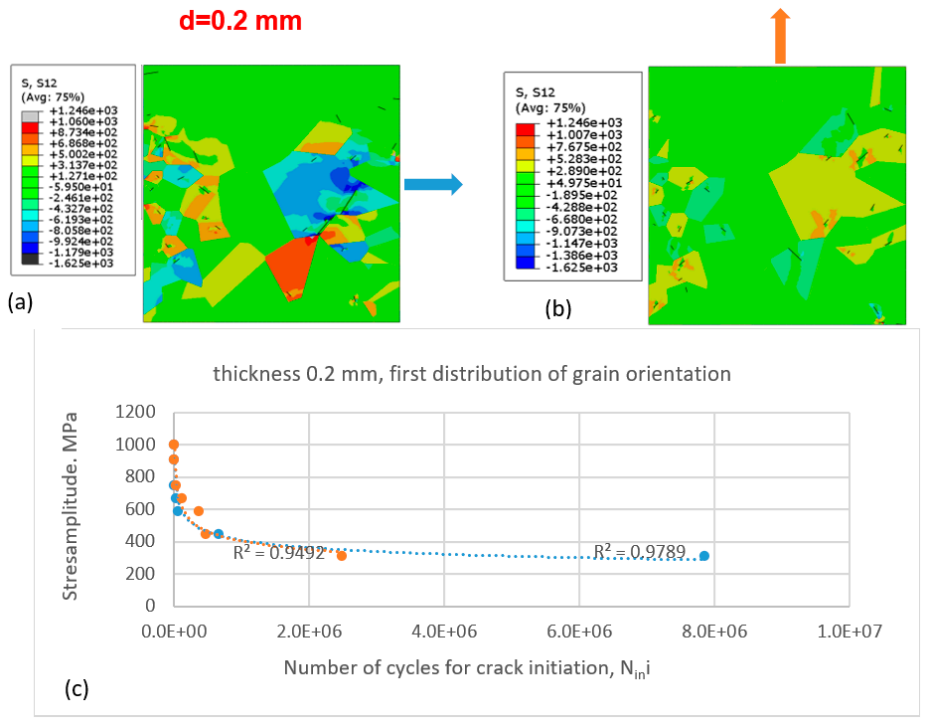
Initiation of fatigue cracks in the cut-out of the microstructure with the first distribution of grain orientation according to Figure 6a under cyclic tensile loading conditions for specimen thickness of 0.2 mm: (**a**) shear-stress distribution and initiated cracks in the cut-out loaded in the x-direction and (**b**) in the y-direction; (**c**) Wöhler diagram for crack initiation for cases (**a**) (blue dashed line and filled circles—loading in the x-direction) and (**b**) (light-orange dashed line and filled circles—in the y-direction).

The same approach was applied for other thickness values of the specimen, d = 0.2 mm (Figure 9).

**Figure 10:**
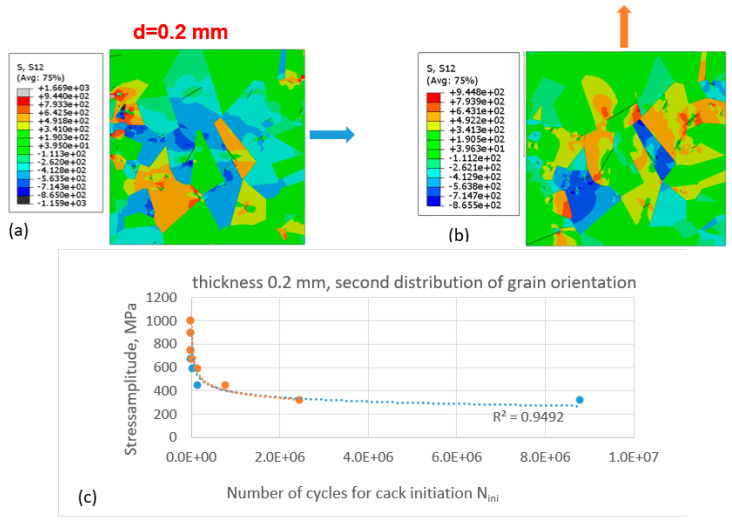
Initiation of fatigue cracks in the cut-out of the microstructure with the second distribution of grain orientation under cyclic tensile loading conditions and for specimen thickness of 0.2 mm: (**a**) shear-stress distribution and initiated cracks in the cut-out loaded in the x-direction; (**b**) shear-stress distribution and initiated cracks in the cut-out loaded in the y-direction; (**c**) Wöhler diagram for crack initiation for cases (**a**) (blue dashed line and filled circles—loading in the x-direction) and (**b**) (light-orange dashed line and filled circles—in the y-direction).

As can be seen in Figure 10a,b, the level of shear-stress distribution is lower compared with the first two cases (with the thickness of the specimen being equal to 0.06 mm).

Different shear-stress distributions and different cracks are observed in Figure 10a,b.

In both figures (Figure 9 and Figure 10), for this thickness value of the specimen, the difference in the number of cycles for lower values of stress amplitude in the Wöhler diagram is clearly pronounced for two different directions of the applied load. In the case of loading in the y-direction (light-orange, filled circles), more grains are favourably oriented due to the formation of persistent slip bands along which the fatigue cracks are formed; therefore, fewer cycles are necessary for the crack to be formed, which is in accordance with the TM model.

### 3.2. FEM Simulations of Crack Initiation in Oligocrystalline Microstructure under Three-Point-Bending Loading Conditions

In the scientific literature, boundary loading conditions in stents are often considered by taking into account bending [30,31] in the first place. It is connected with the fact that bending is the one of the major mechanisms of loading in stents under real conditions. A stent is subjected to the action of different loading types, such as pressure, bending, tensile loading, and compression. However, bending can cause significant effects on the behaviour of the stent, especially under the condition of severe deformation and contact with the vessel wall. Therefore, a great body of research deals with bending loading conditions. This would help to understand how stents (and materials) reply to loads and how the construction process and materials should be changed in order to improve the stent life cycle.

**Figure 11:**
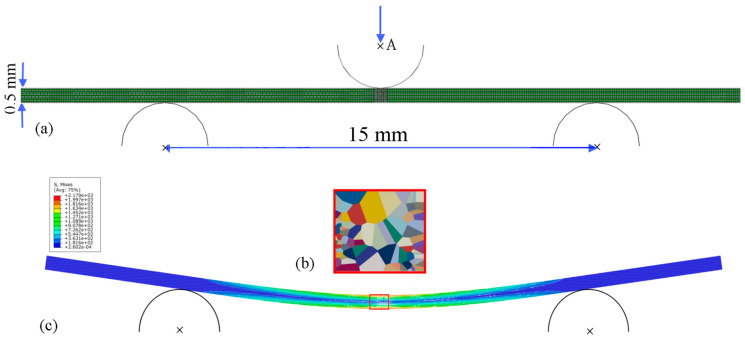
(**a**) FE mesh and boundary conditions applied to the specimen; (**b**) cut-out of a piece of the beam; (**c**) the beam after bending, with Mises stress distribution, x- is the reference points of the pins.

FE simulations of fatigue crack initiation in the cut-out of the microstructure under cyclic three-point-bending loading conditions were performed with the application of the submodelling approach. First, a 3D macromodel with the dimensions of the beam matching the one tested in the experiment was created (Figure 11a); then, a cut-out of the microstructure (Figure 11b) was inserted in the central part of the beam under the pin. The simulations were performed under cyclic displacement loading conditions.

The finite element discretization is depicted in Figure 11a, where 14,800 C3D8 linear elements were applied to the beam outside the microstructural cut-out, and a total of 3592 linear membrane elements (M3D4R and M3D3) were applied to the cut-out of the microstructure.

This FE model was 25 mm long and 0.5 mm wide, and two values of beam thickness were considered: d = 0.01 mm and d = 0.2 mm. The simulations were performed for the two grain-orientation distributions (as in Figure 6a,b).

Since displacement boundary conditions tend to be more stable than force boundary conditions when modelling contact problems in ABAQUS and convergence is easier to achieve, the displacement-controlled load was applied at Point A in such a way that the surface of the pin followed Point A. The displacement values were chosen in such a manner that the level of stress inside the cut-out of the microstructure was comparable to the stress obtained in the case of cyclic tensile loading simulations (Section 3.1). When deflecting two beams with different thickness to achieve the same deflection, it is expected that the thicker beam requires greater force.

**Figure 12:**
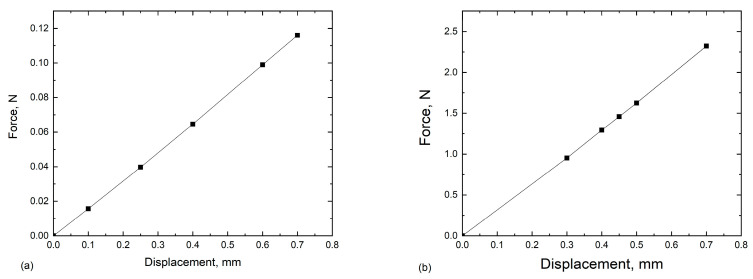
Dependence of the force applied to the pin on displacemen for two thickness values of the beam: (**a**) 0.01 mm and (**b**) 0.2 mm.

The corresponding achievable loads for the different displacement values applied to the pin are presented in Figure 12 for two thickness values of the beam: (a) 0.01 mm and (b) 0.2 mm. It is clear that the loads are proportional to the thickness of the beam.

The ENCASTRE boundary conditions were imposed on the reference points of the supporting pins of the beam, and the displacement boundary condition was applied to the reference point of the upper pin. The displacement values were chosen in such a manner that the reasonable force was applied to the beam, and the corresponding stress amplitude was determined following the formula for flexural applied stress [32]:

(2) $$\sigma =\frac{3PL}{2w^{2}d}.$$

where *P* is the force applied to the upper pin, *L* is the span between two supporting pins, *w* is the width of the cantilever beam, and *d* is the thickness of the beam.

The stress amplitude applied to the beam, taking into account the thickness of the beam equal to *d* = 0.01 mm, was calculated according to the following formula:

(3) $$\sigma =\frac{3PL}{2w^{2}}=9000*\frac{P}{{mm}^{2}},\;\mathrm{thickness}=0.01\mathrm{mm}$$

The coefficient, 9000, before the load in the formula was obtained by taking into account the geometry of the specimen: the span of the supporting pins was *L* = 15 mm; width of the specimen *w* = 0.5 mm; and thickness *d* = 0.01 mm.

For the thickness of the beam of 0.2, the following formula was obtained after inserting thickness of 0.2 instead of 0.01 as in the first case:

(4) $$\sigma =\frac{3PL}{2w^{2}}=450*\frac{P}{{mm}^{2}},\;\mathrm{thickness}=0.2\mathrm{mm}$$

The corresponding values of displacement, force, and stress for both thickness values of the beam are listed in Table 3.

As can be seen from the Table 4, with the increase in thickness, the force increased, also to achieve the prescribed value of displacement, and the calculated stress amplitudes also lie within the same range.

**Figure 13:**
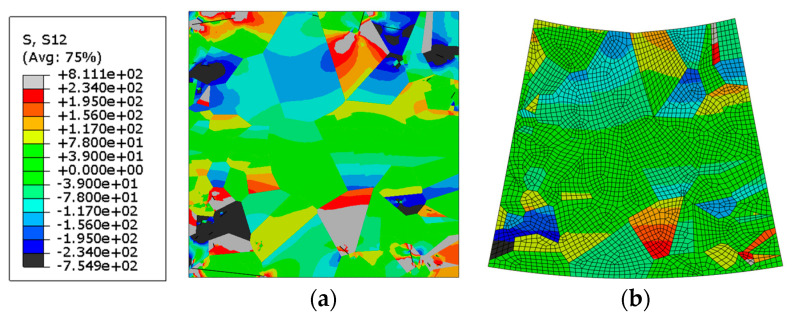
(**a**) Shear-stress distribution in the cut-out of the microstructure under cyclic bending loading conditions with initiated cracks; (**b**) deformed shape of the cut-out of microstructure (×20 magnification).

The contour plot of shear-stress distribution in the cut-out of the microstructure and the FE mesh on the deformed piece of microstructure under cyclic three-point-bending loading conditions are shown in Figure 13a,b, respectively. The areas of the grains where the shear stress exceeds the critical resolved shear stress are indicated by grey and black colours. Persistent slip bands are expected to develop in these areas with the following fatigue crack initiation along the predefined shear bands.

**Figure 14:**
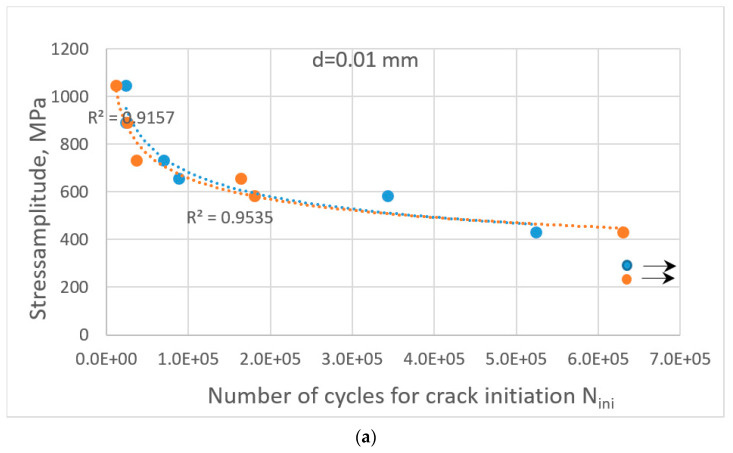

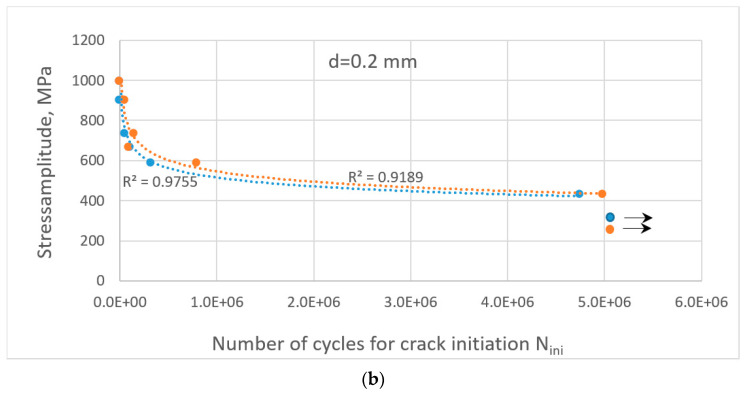
Wöhler diagrams for crack initiation in the case of three-point-bending loading conditions for two thickness values of the specimen: d = (**a**) 0.01 mm; (**b**) 0.2 mm. Different colours depict different distributions of grain orientation in the same cut-out of the microstructure.

One can also notice the existence of a neutral zone in the bending specimen, where the stress values are lower than the critical ones to cause cracking along the shear bands. Therefore, the cracks in the bending specimen are observed in the upper (compression) region and lower (tensile) region of the specimen. Depending on the grain sizes in the active slipping zones in the bending specimen, it can cause an increase in the number of cycles or a decrease in it in comparison with the case of tensile loading conditions. In our particular case (with smaller grains in the active zones), this resulted in an increase in the number of cycles for crack initiation. This resulted in a higher number of cycles at lower amplitude (marked with arrows in Figure 14a,b).

The resulting Wöhler diagrams for crack initiation for two thickness values of the specimen and different distributions of grain orientation are depicted in Figure 14.

A comparison of the curves for crack initiation in the cut-out of the microstructure with the same thickness and different grain-orientation distributions shows the results below (Figure 14a,b).

In spite of the similarity in the shape of the trending lines of the Wöhler curves for crack initiation, in both cases, they have some scattering in the number of cycles. Both curves are seen to have two segments with different slopes: the first one in the region of higher applied stress amplitude and the second one practically horizontal for applied stress less than 400 MPa. As can be observed from the figure, more scattering in the number of cycles for crack initiation for two distributions of grain orientation is observed for the thickness of 0.2. For the thickness of 0.01 mm, some points even coincide for both grain-orientation distributions.

During the last decades, a lot of models for fatigue crack initiation have been elaborated [33]. What is also clear is that any predictive technique must be based on the full knowledge of the key microstructural features that may exist and remain important over a range of differing lengths. The research literature dedicated to fatigue crack nucleation addresses the following issues: slip localization and persistent slip bands, grain boundaries, slip transfer and interfaces, micro-texture and twins, nucleation criteria, and cracks. Among all the most generic modelling techniques relevant to fatigue modelling covering scales from atomistic (10^−9^ m) to continuum (10^0^ m) lengths are molecular dynamics, discrete dislocation plasticity, crystal plasticity, and conventional (Mises) continuum plasticity.

While crystal-plasticity modelling, based on models by Taylor and Sachs, has significantly contributed to our understanding of fatigue crack initiation, it does have some drawbacks that can limit its predictive capabilities. The necessity to model the behaviour of individual crystals within the microstructure results in an increase in computational complexity, making it challenging to simulate large-scale structures or complex loading conditions. Crystal-plasticity models incorporate crystallographic orientation and grain interactions at a more detailed level than this computational model based on the Tanaka–Mura approach.

The proposed model can be considered a simplified crystal-plasticity approach predicting crack initiation in a material, explicitly taking the microstructure into consideration. The advantage of this model is the ease of its implementation, applicability to different materials, experimental validation.

At present, there is some room for the development of this model. It only considers crack initiation in the interior of the grains, without considering crack initiation at the grain boundaries. The orientation of the grains considered in the model is restricted to 2D cases, and there is no direct way to input a real microstructure in the model. These limitations can be overcome in our future work.

## 4. Conclusions

The following conclusions can be drawn from the results obtained:

- Predictions of the number of cycles for fatigue crack initiation in the cut-out of an oligocrystalline microstructure resembling the one observed in the experiment were performed using FEM analysis with the application of the physically based Tanaka–Mura model.
- Simulations of fatigue crack initiation in the cut-out of the microstructure were performed under cyclic tensile and bending loading conditions. For the case of three-point-bending loading conditions, the submodelling approach within the framework of FEM simulations was applied.
- Wöhler diagrams for fatigue crack initiation in the cut-out of the microstructure were constructed. The effect of the type of loading conditions, thickness of the specimen, and different grain orientation on the Wöhler diagrams for crack initiation were analysed.
- It was found that the microstructural features of oligocrystals are very sensitive to different loading conditions with respect to their fatigue behaviour and play a major role in fatigue crack initiation.
- Larger thickness of the specimen results in an increase in the number of cycles for crack initiation.
- The presence of a neutral zone in the bending specimen results in an increase in the number of cycles in our specific case (with small grains in active zones).
- The presented approach developed in this contribution can serve as a framework for the future design of stent components.

## Figures and Tables

**Table 1:** Chemical composition of 316 LVM stainless steel.

Element	C	Si	Mn	P	S	N	Cr	Mo	Ni	Cu	Fe
Max%	0.03	1.00	2.00	0.025	0.010	0.1	19.00	3.5	15.00	0.5	-

**Table 2:** Physical properties of 316 LVM stainless steel.

Density	8 g/cm^3^
Melting point	1500 °C
Coefficient of expansion	16.5 1/°C
Modulus of rigidity	70.3 kN/mm^2^
Modulus of elasticity	187.5 kN/mm^2^

**Table 3:** Parameters of the model.

*G*	W_c_	CRSS
70,300 MPa	69 N/mm	117 MPa

**Table 4:** Displacement, force, and stress amplitude for two thickness values of the beam.

Displacement, mm	Thickness of 0.01 mm		Thickness of 0.2 mm	
	Force, N	Stress, MPa	Force, N	Stress, MPa
0.3	−0.047	429.17	−0.9536	429.12
0.4	−0.064	582.26	−1.2937	582.19
0.45	−0.0739	656.23	−1.4582	656.19
0.5	−0.0811	730.28	−1.6253	731
0.6	−0.0990	891	−1.9791	890.59
0.7	−0.1161	990	−2.3216	1044.72

## Data Availability

Will be available upon request.
